# Insertion of Plate Teeth, on a Pivot

**Published:** 1853-01

**Authors:** S. A. Saltenstall

**Affiliations:** Dentist, Columbus, Mi.


### ARTICLE XI.

*Insertion of Plate Teeth on a Pivot.* By S. A. SALTENSTALL, Dentist, Columbus, Mi.

MESSRS. EDITORS: Enclosed I send you a specimen of my mode of inserting plate teeth on pivot.

In the first place, I prepare the fang in the usual way, by cutting off the tooth and perforating the fang as deep as practicable. I then introduce a hollow gold wire that will fit neatly and arranged so as to be readily removed. After the hollow wire is adjusted I fit a plate to the fang and perforate the plate sufficiently to admit the hollow wire to pass through it; then take common beeswax and press over the plate and wire and remove the whole and secure it in plaster, taking care to retain the position. When the plaster is sufficiently dried, remove the wax, and solder the two together. The plate that covers the fang should be a little longer than necessary on the inside and leave room to bend the plate and perforate it near the base. Then fit it to the fang and select a tooth of the proper size and shade, adjust it to fit the plate, plate the tooth and leave the back sufficiently long to cover the base and pass through the perforation in the base, after the plate is arranged to give the tooth its proper place in the mouth, let the point of the backing pass through the perforation in the base, then grasp the two points of the plates on the inside with a pair of small pliers, remove the whole and cover the tooth and wire with plaster, when dry, solder the two together, cut one-sixteenth of an inch off the hollow wire, then finish by polishing the gold. This done, introduce a well seasoned hickory pivot into the hollow wire and force it up as far as the hollow extends, leaving the hickory one-sixteenth of an inch longer than the gold, the exposed one-sixteenth of an inch of hickory must be the full size of the hollow wire. Then wrap the full length of the pivot in several folds of gold foil and force it to its place in the fang, and the operation is completed.

I have adopted this method in several instances where the irregularity of the natural teeth approximated deformity, and succeeded in giving the artificial teeth a perfectly symmetrical appearance. I have tested this mode to my entire satisfaction, and believe it to be superior to any other mode hitherto made public.

---

